# Online registration of neonatal stroke in Shenzhen: protocol for a multicentre, prospective, observational cohort study

**DOI:** 10.3389/fped.2026.1775052

**Published:** 2026-05-08

**Authors:** Xiaoli Zhao, Hongqin Zhang, Lijuan Sheng, Zhangbin Yu, Yanping Guo, Hanni Lin, Xuelei Gong, Xueli Zhang, Miao Tang, Jiaoyu Mao, Genshen Zheng, YuanYuan Li, Yan Huang, Fangshi Zhang, Zhangcong Liang, Jing Feng, Lichun Bai, Yaping Liu, Yinsha Cai, Zhifeng Huang, Qianshen Zhang, Aiyun Zhang, Xiaoyi Fang, Jingxing Cai, Xue Tang, Xufeng Luo, Jinxing Feng, Lina Men, Di Gao

**Affiliations:** 1 Department of Neonatology, Shenzhen Children's Hospital, Shenzhen, Guangdong, China; 2 Department of Neonatology, Nanshan Maternal and Child Healthcare Hospital, Shenzhen, Guangdong, China; 3 Department of Neonatology, Shenzhen People's Hospital, Shenzhen, Guangdong, China; 4 Department of Neonatology, Peking University Shenzhen Hospital, Shenzhen, Guangdong, China; 5 Department of Neonatology, Shenzhen Luohu People's Hospital, Shenzhen, Guangdong, China; 6 Department of Neonatology, Shenzhen Luohu Maternal and Child Healthcare Hospital, Shenzhen, Guangdong, China; 7 Department of Neonatology, Longhua People's Hospital, Shenzhen, Guangdong, China; 8 Department of Neonatology, Futian District Maternal and Child Health Hospital, Shenzhen, Guangdong, China; 9 Department of Neonatology, Shenzhen Nanshan People's Hospital, Shenzhen, Guangdong, China; 10 Department of Neonatology, Shenzhen Far East Obstetrics and Gynecology Hospital, Longgang Obstetrics and Gynecology Hospital, Shenzhen, Guangdong, China; 11 Department of Neonatology, Shenzhen Bao'an People's Hospital, Shenzhen, Guangdong, China; 12 Department of Neonatology, Shenzhen Baoan Women's and Children's Hospital, Shenzhen, Guangdong, China; 13 Department of Neonatology, Shenzhen Yantian District People's Hospital, Shenzhen, Guangdong, China; 14 Department of Neonatology, Shenzhen Longgang District Third People's Hospital, Guangdong, Shenzhen, China; 15 Department of Neonatology, Longgang District Maternal and Child Healthcare Hospital, Shenzhen, Guangdong, China; 16 Department of Neonatology, Shenzhen Guangming District People's Hospital, Shenzhen, Guangdong, China; 17 Department of Neonatology, Shenzhen Third People's Hospital, Shenzhen, Guangdong, China; 18 Department of Neonatology, Shenzhen Second People's Hospital, Shenzhen, Guangdong, China; 19 Department of Neonatology, Shenzhen Maternal and Child Healthcare Hospital, Shenzhen, Guangdong, China; 20 Department of Neonatology, The University of Hong Kong-Shenzhen Hospital, Shenzhen, Guangdong, China; 21 Department of Neonatology, The Seventh Affiliated Hospital of Sun Yat-sen University, Shenzhen, Guangdong, China; 22 Department of Neonatology, The Eighth Affiliated Hospital of Sun Yat-sen University, Shenzhen, Guangdong, China

**Keywords:** follow-up strategy, infant, neurodevelopmental disorder, registry, stroke

## Abstract

**Introduction:**

Neonatal stroke remains a primary cause of permanent neurological disability. While global prognostic frameworks, including magnetic resonance imaging (MRI) of the posterior limb of the internal capsule (PLIC), General Movements Assessment (GMA), and the Hammersmith Infant Neurological Examination (HINE), have been established to predict motor outcomes, these tools have not been systematically integrated into large-scale Chinese clinical registries. In regions like Shenzhen, the scarcity of prospective, multicenter data continues to hinder the characterization of stroke incidence, risk factors, and long-term outcomes. This protocol details the establishment of the first dedicated online registry in Shenzhen to address these critical evidence gaps.

**Methods and analysis:**

This multicenter, prospective, observational cohort study aims to enroll 300 neonates diagnosed with stroke, including arterial ischemic, hemorrhagic, and cerebral venous sinus thrombosis subtypes from a network of 22 hospitals in Shenzhen, China, between January 2025 and December 2030. Data collection will utilize standardized electronic case report forms (eCRFs), initially managed via a uniform digital system ( Supplementary File 1), with a planned transition to a secure web-based registry for broader access. Participants will undergo longitudinal follow-up until age five, with neurodevelopmental outcomes assessed using the Bayley Scales of Infant and Toddler Development (4th Ed., Chinese Version), 3T cranial MRI, and video-EEG. To explore the metabolic-inflammatory nexus of neonatal injury, placental pathology will be integrated into the clinical data, and a biobank of stool and breast milk samples will be established. The primary outcome is the registry-based incidence of neonatal stroke. In contrast, secondary outcomes include characterizing clinical subtypes, identifying prenatal/perinatal risk factors, and developing and internally validating a robust prognostic prediction model.

**Clinical Trial Registration:** https://www.chictr.org.cn, identifier ChiCTR2500097104.

## 1 Introduction

Neonatal stroke, defined as an acute focal neurological injury secondary to arterial ischemic infarction, hemorrhagic stroke, or cerebral venous sinus thrombosis occurring between birth and 28 days of life, is a leading cause of permanent neurological disability in infants (1, 2). This cerebrovascular event results in lifelong sequelae such as cerebral palsy, epilepsy, and cognitive impairments, with an estimated incidence ranging from 1 in 1,100 to 1 in 4,000 live births for perinatal stroke; the incidence of strictly neonatal stroke is less well defined but is approximately 1 in 3,000 to 1 in 5,000 live births (3–5) Critically, mortality rates range from 4% to 10%, underscoring its Status as a primary pediatric health concern (6, 7). Despite its profound impact, evidence-based strategies for prevention, acute management, and long-term prognostication remain significantly underdeveloped, with no standardized, population-based surveillance and outcome-tracking systems in many regions.

A central barrier to improving care is the profound lack of prospective, high-quality data on disease epidemiology and natural history (8). While countries such as the United Kingdom, Canada, and Australia have established national neonatal stroke registries to provide continuous, standardized monitoring of aetiology and outcomes (9, 10), such systematic data-collection initiatives are absent in China. Several other countries have successfully developed neonatal and infantile thrombosis registries; for example, the Italian Registry of Infantile Thrombosis (RITI) has provided valuable lessons regarding multicenter collaboration, data standardization, and the challenges of prospective data collection (9). Fragmented, retrospective studies and significant discrepancies in reported incidence rates characterize the existing evidence. For instance, a multicenter study in Beijing reported a rate of 1 in 2,660 (10), whereas a retrospective review across Shenzhen reported a much lower rate of 1 in 8,373 (10). These discrepancies likely reflect differences in case ascertainment (active surveillance vs. retrospective chart review), imaging protocols (universal MRI vs. selective cranial ultrasound), and referral patterns to tertiary centres, rather than true geographic variation in disease frequency. Furthermore, the long-term neurodevelopmental trajectory for survivors, a critical endpoint for families and healthcare systems, is poorly documented, and clinical management, especially for hemorrhagic and venous stroke subtypes, lacks consensus guidelines, leading to potentially inconsistent care.

Prognostication of motor outcome following neonatal stroke has been an active area of research. Magnetic resonance imaging (MRI) assessment of the posterior limb of the internal capsule (PLIC) has been shown to correlate with the development of hemiparesis (11, 12). Quantitative scoring systems such as the Modified Pediatric ASPECTS have also been developed to correlate infarct volume with clinical outcomes (13). Additionally, the Hammersmith Infant Neurological Examination (HINE) and general movements evaluation are validated tools for early prediction of cerebral palsy (12). We acknowledge that this registry enrols only diagnosed cases; therefore, cumulative incidence will be calculated using total live births from all 22 participating hospitals as the denominator. However, this represents a hospital-based cohort rather than true population-based surveillance, and referral patterns may bias incidence estimates.

Shenzhen is particularly suitable as a model region due to its annual birth rate of approximately 200,000 live births, >98% NICU coverage across all districts, universal availability of cranial MRI and video-EEG at all participating hospitals, and the fact that >95% of regional births occur within the 22 study hospitals.

Therefore, establishing a prospective, multicenter cohort and dedicated registry is an urgent and foundational step. Such a resource is essential for accurately defining the disease burden, identifying associations with prenatal and perinatal factors that may inform future interventional studies, and mapping the evolution of neurodevelopmental outcomes from infancy through early childhood. By capturing comprehensive, longitudinal data within a real-world clinical setting, a registry can bridge the critical evidence gap between isolated clinical observations and population-level understanding.

To address this unmet need, we have designed the Shenzhen Neonatal Stroke Online Registry. In this study, we hypothesize that implementing a standardized, multicenter, prospective cohort will yield accurate epidemiological data, identify key risk factors associated with adverse outcomes, and establish a robust platform for developing prognostic models. We present the protocol for a six-year, prospective, observational cohort study involving 22 hospitals in Shenzhen. The primary objectives are to determine the registry-based incidence of neonatal stroke and to evaluate comprehensive neurodevelopmental outcomes at 5 years of age. Secondary objectives include characterizing stroke subtypes, analyzing current management disparities, and integrating a biobank for future mechanistic research. This protocol aims to provide a replicable model for disease surveillance and to generate high-quality evidence to inform clinical guidelines, optimize care pathways, and ultimately improve long-term outcomes for infants affected by stroke.

## 2 Methods and analysis

This study protocol has been developed in accordance with the Standard Protocol Items: Recommendations for Interventional Trials (SPIRIT) guidelines, adapted for an observational cohort study.

### 2.1 Study objectives

#### 2.1.1 Primary objective

To determine registry-based incidences of neonatal stroke in the Shenzhen region and to evaluate the long-term neurodevelopmental outcomes of affected children at age 5.

#### 2.1.2 Secondary objectives

1. To characterize the clinical presentation, short-term treatment efficacy, and safety of the three subtypes of neonatal stroke: arterial ischemic stroke, cerebral sinovenous thrombosis, and hemorrhagic stroke.
2. To identify significant prenatal, perinatal, and postnatal risk factors for neonatal stroke, and to develop and validate predictive models for risk stratification using a panel of early biochemical and imaging biomarkers.
3. To analyze regional and institutional variations in the diagnostic, therapeutic, and rehabilitative management of neonatal stroke, and to propose evidence-based strategies for optimizing care and standardizing treatment protocols.

### 2.2 Study design

This multicenter, prospective, observational cohort study aims to investigate the long-term neurodevelopmental outcomes of neonates with stroke. The study is planned to run for six years, from January 2025 to December 2030, in Shenzhen, China. The initial network comprises 22 collaborating hospitals, with Shenzhen Children's Hospital as the lead institution and Shenzhen People's Hospital as the primary coordinating center.

**Figure 1:**
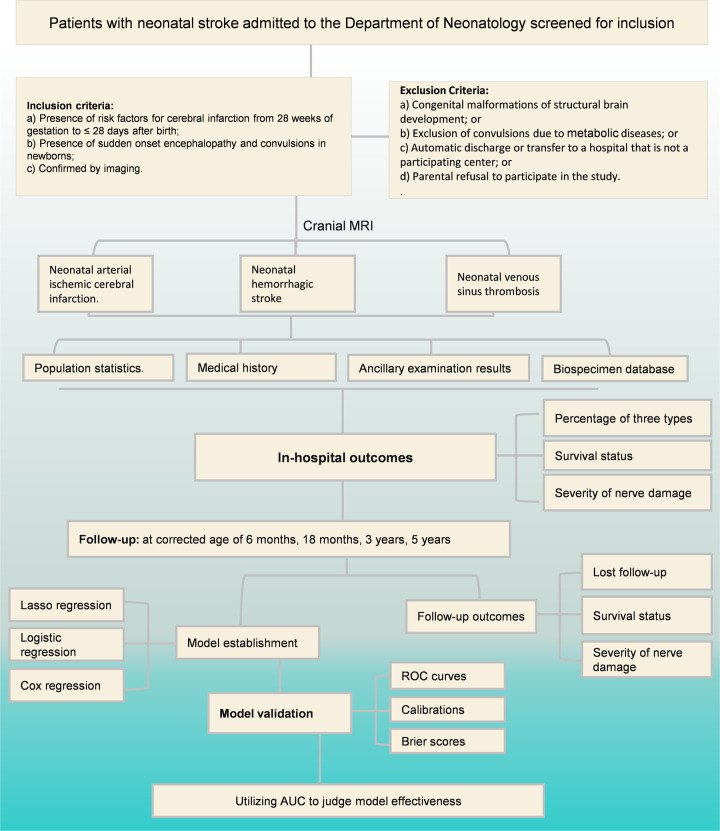
Study Flowchart: Clinical Features, Management, and Outcomes of Neonatal Stroke.

The study aims to recruit 300 neonates diagnosed with neonatal stroke, following them prospectively for five years to assess their neurodevelopmental trajectory. Additional hospitals may be included throughout the study period. A detailed study flow diagram is presented in Figure 1.

**Figure 2:**
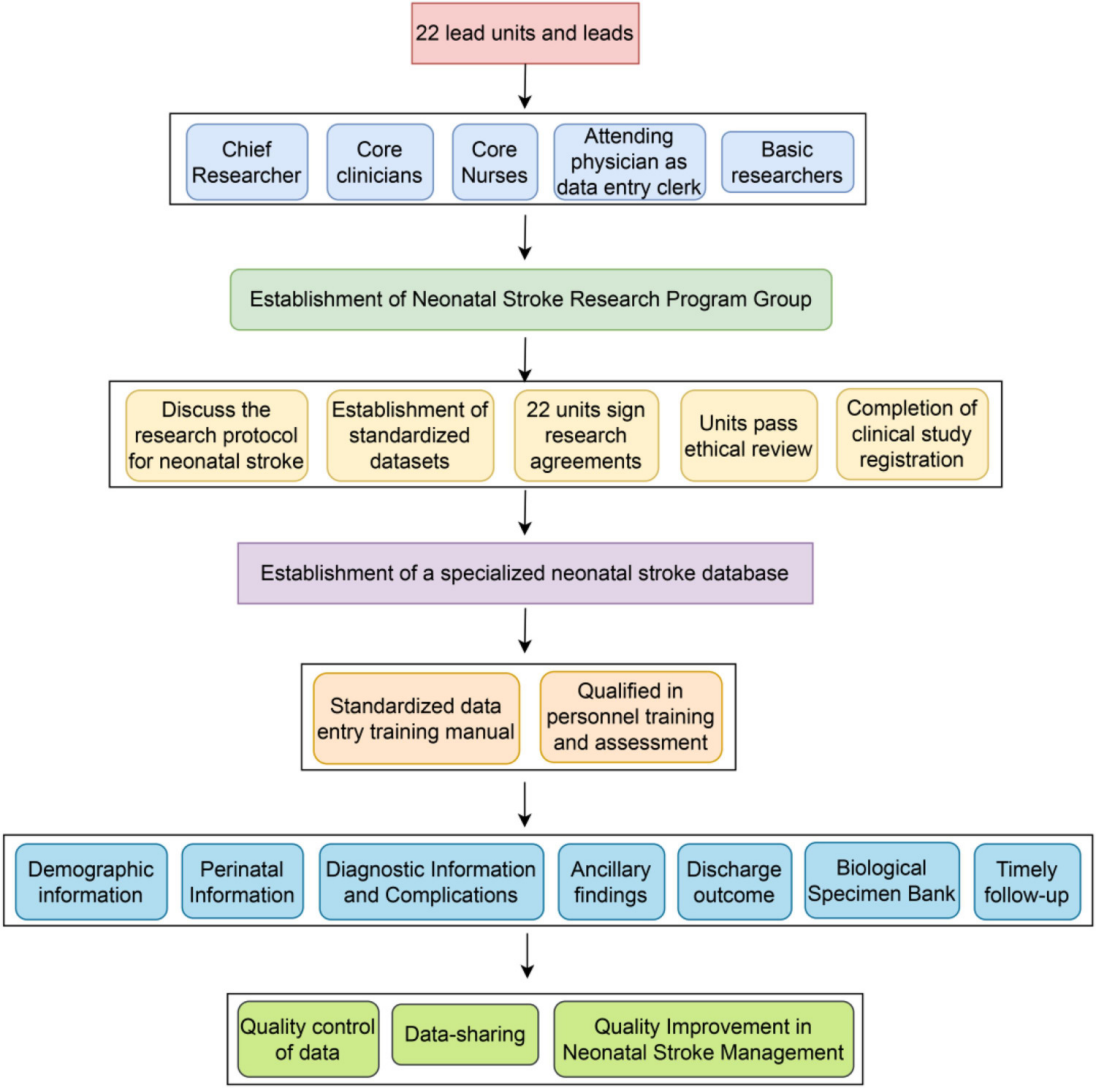
Schematic of the multi-phase neonatal stroke research program infrastructure.

### 2.3 Study participants

#### 2.3.1 Inclusion criteria

Participants must meet all the following criteria:

1. Gestational age ≥28 weeks and ≤28 days postnatal, with identified stroke risk factors. (Extremely preterm infants <28 weeks are excluded by design due to diagnostic challenges and different pathophysiology.
2. Clinical presentation of acute encephalopathy or seizures.
3. Neuroimaging confirmation (MRI) of stroke (arterial ischemic, hemorrhagic, or cerebral venous sinus thrombosis), excluding other common etiologies. Clinically silent infarctions (incidental findings on imaging without clinical symptoms) will be excluded. To ensure complete case ascertainment, all neonates with identified risk factors for stroke (e.g., congenital heart disease, extracorporeal membrane oxygenation, or severe perinatal asphyxia) will undergo systematic screening with cranial MRI, and any asymptomatic infarctions detected will be documented separately but excluded from the primary analysis cohort**.**
4. Parental or legal guardian consent.

#### 2.3.2 Exclusion criteria

Participants will be excluded for any of the following:

1. Congenital structural brain malformations.
2. Seizures attributable to a metabolic cause (e.g., electrolyte imbalances or hypoglycemia).
3. Transfer to a non-participating center or voluntary discharge prior to enrollment.
4. Refusal of informed consent by the parent or guardian.

#### 2.3.3 Withdrawal criteria

Participants will be withdrawn from the study if:

1. The participant or legal guardian withdraws consent.
2. The participant fails to complete scheduled follow-up assessments.
3. Clinical deterioration renders continued participation unsafe or impractical.

#### 2.3.4 Elimination criteria

Participants will be eliminated from the final analysis if:

1. No data is recorded throughout the study.
2. Serious protocol deviations occur (e.g., unapproved concomitant therapies and noncompliance with key procedures).
3. Medications or interventions that could confound results are administered during the study.

### 2.4 Study procedures

#### 2.4.1 Recruitment, screening, and informed consent

Neonates meeting the inclusion criteria will be prospectively identified and recruited by trained neonatologists from the NICUs and pediatric wards of the 22 participating hospitals. Two attending neonatologists at each site will be responsible for patient enrollment using standardized diagnostic criteria.

Written informed consent will be obtained from the parents or legal guardians after a definitive diagnosis is made. The study adheres to the ethical principles of the Declaration of Helsinki and has received institutional ethics approval.

To ensure rigorous standardization across the 22 participating centres, all enrolling neonatologists will undergo a mandatory centralized training workshop focused on standardized case definitions, MRI interpretation using a validated study atlas, and EEG acquisition protocols. In parallel, to maintain longitudinal data quality, annual inter-rater reliability assessments will be conducted using 10 blinded test cases. For diagnostic adjudication, all stroke subtypes will be centrally reviewed by an expert committee comprising three senior neonatologists and two neuroradiologists at Shenzhen Children's Hospital, who remain blinded to the initial site-level diagnosis. Furthermore, neuroimaging will adhere to a harmonized 3T MRI protocol (including DWI, SWI, and MRA/MRV sequences). Where 3T MRI is not immediately available at a participating center, arrangements will be made for patient transfer to a center with 3T capacity, and this will be documented. Specific scoring approaches for infarct volume (modified Pediatric ASPECTS) and PLIC assessment will be used centrally. All video-EEG recordings will be centrally interpreted by a single epileptologist masked to all clinical and imaging data.

#### 2.4.2 Data collection, management, and online registry

Data will be collected using a standardized Excel data collection form (provided as Supplementary File 1). A secure, web-based electronic registry is planned for future public access and multicenter expansion. The registry will capture both inpatient and post-discharge follow-up data, manually entered by trained neonatal attending physicians at each center.

- Data Entry and Management: Each participant will be assigned a unique ID. Baseline data (demographics, medical history, MRI scans, EEG, and coagulation function) will be recorded within 24 h of diagnosis.
- **Follow-up Data Collection**: Follow-up assessments will be conducted at 6 months, 18 months, 3 years, and 5 years (corrected age). The selection of these time points was based on published literature and local clinical practice in China: 6 months is a key age for motor assessment and early intervention (routine well-child visit); 3 years allows comprehensive cognitive, language, and behavioral assessment (preschool entry examination); and 5 years permits assessment of learning disabilities, attention problems, advanced cognitive functions, and social adaptation (preschool exit/primary school entry) (14, 15). Follow-ups can be done at any participating center based on patient preference.
- Retention Strategies: To encourage follow-up, commemorative gifts will be given on Children's Day (June 1) and patients’ birthdays. For families facing financial hardship, follow-up costs will be covered by the Neonatal Department's funding at Shenzhen Children's Hospital.
- Governance: A Clinical Research Data Monitoring Committee (chaired by Di Gao) will oversee protocol compliance, training, progress reviews, data analysis, and reporting.

#### 2.4.3 Data elements and database modules

The online registry is divided into the following modules:

1. General and Hospital Information: Demographics, birth facility data, and administrative details.
2. Family and Perinatal History: Family history, maternal/pregnancy data, labor/delivery details, and resuscitation history.
3. Neonatal Clinical Presentation: Initial Status (e.g., blood glucose), seizure data, and comorbidities.
4. Diagnostic Tests and Biological Samples: Coagulation tests, genetic data (optional), neuroimaging, and EEG results.
5. Treatment and Discharge Summary: Details of therapies, discharge diagnosis, and treatment outcomes.
6. Long-Term Follow-up Examinations: Data from 6m, 18m, 3y, and 5y visits, including Bayley-4C scores, EEG, MRI results, growth metrics, and epilepsy medication.
7. Follow-up Conclusions: Final Status, including outcomes like epilepsy, cognitive impairment, motor disorders, and cerebral palsy, confirmed by pediatric neurologists and rehabilitation physicians.

The complete data dictionary is available in Online Supplementary File 3.

#### 2.4.4 Standardized diagnostic and outcome criteria

Key research variables are defined as follows:

- Neonatal Arterial Ischemic Cerebral Infarction: Ischemic necrosis caused by cerebral artery infarction in newborns.
- Neonatal Hemorrhagic Stroke: Intracranial or intraventricular hemorrhage in term infants (37–42 weeks GA) within 28 days.
- Neonatal Cerebral Venous Sinus Thrombosis: Thrombotic occlusion of venous sinuses or cerebral veins.
- Neonatal Seizures: Abnormal EEG activity with stereotypical, episodic manifestations.
- Perinatal Maternal Diseases: Includes gestational diabetes mellitus and hypertension.
- Congenital Heart Disease: Structural anomalies of the heart or major vessels.
- Neonatal Polycythemia: Venous hematocrit ≥65% in the first week.
- Twin-to-Twin Transfusion Syndrome: Significant hemoglobin and weight differences between twins.
- Disseminated Intravascular Coagulation: Systemic coagulation activation syndrome.
- Early-onset Neonatal Sepsis: Sepsis onset within 72 h of life.
- Neonatal Hypoxic-Ischemic Encephalopathy: Brain damage from perinatal asphyxia.
- Neonatal Hypoglycemia and Hyperglycemia: Blood glucose < 2.2 mmol/L and > 7 mmol/L, respectively.
- Bayley Scales of Infant and Toddler Development (Bayley-4C): At all follow-up time points (6 months, 18 months, 3 years, and 5 years), we use the Chinese version of the Bayley Scales of Infant and Toddler Development, Fourth Edition (Bayley-4C) as the sole standardized instrument. Other validated instruments, such as the Hammersmith Infant Neurological Examination (HINE) and Griffiths Mental Development Scales, are not used in the current protocol but may be considered in future extensions.

#### 2.4.5 Biobanking procedures

The primary mechanistic hypothesis is that gut dysbiosis and/or breast milk composition modulate neuroinflammatory pathways and long-term neurodevelopmental outcomes. Planned analyses include 16S rRNA microbiome profiling and targeted cytokine/chemokine assays (e.g., IL-6, IL-1β, TNF-α).

Biological samples will be collected three times: within 48 h of diagnosis, at 7 days post-diagnosis, and prior to discharge.

- Samples: 5–8 grams of stool and 3–5 mL of breast milk.
- Procedure: Samples will be stored initially at −20 °C at the collection site and transferred to a centralized −80 °C freezer at Shenzhen Children's Hospital for long-term storage.

Long-term storage governance: samples will be stored for up to 10 years. Access requires approval by a Biobank Access Committee. Separate informed consent will be obtained for any future genetic or exploratory analyses beyond the primary aims. As a constructive addition, residual serum from routine clinical blood draws will be collected for exploratory analysis of neuronal injury biomarkers (e.g., S100B).

#### 2.4.6 Long-term follow-up assessment schedule

Follow-up assessments will occur at corrected ages of 6 months, 18 months, 3 years, and 5 years. A schedule detailing the assessments and measures taken at each time point is summarized in Table 1.

**Table 1:** Schedule of long-term follow-up assessments.

Assessment	6 Months	18 Months	3 Years	5 Years
Bayley Scales of Infant and Toddler Development, Fourth Edition, Chinese Version (Bayley-4C)	✓	✓	✓	✓
Growth metrics (weight, length/height, head circumference)	✓	✓	✓	✓
Neurological examination	✓	✓	✓	✓
Seizure/epilepsy assessment	✓	✓	✓	✓
Epilepsy medication review			✓	✓
Video-EEG			✓	✓
Cranial MRI (3T)			As needed	As needed
Cognitive, language, and behavioral assessment			✓	✓
Learning disability assessment				✓
Attention problems assessment				✓
Social adaptation assessment				✓
Final outcome confirmation (cerebral palsy, cognitive impairment, epilepsy)				✓

✓, assessment performed; MRI performed only when clinically indicated. All ages are corrected ages. Bayley-4C, Bayley Scales of Infant and Toddler Development, Fourth Edition, Chinese Version; EEG, electroencephalography; MRI, magnetic resonance imaging.

### 2.5 Outcome measures

#### 2.5.1 Primary outcomes

Registry-based incidence of neonatal strokes in Shenzhen.

#### 2.5.2 Secondary outcomes

- Survival status at hospital discharge and throughout the 5-year follow-up period.
- Neurodevelopmental outcomes at 5 years were defined as a composite based on Bayley-4C scores and clinical diagnoses of impairments (e.g., cerebral palsy, epilepsy).
- Risk factor characterization (prenatal, perinatal, and postnatal).
- Performance of the developed prognostic prediction model for adverse neurodevelopmental outcomes.

### 2.6 Sample size calculation

Assuming a 30% neurodevelopmental outcome rate at 5 years (based on published literature), a final sample of approximately 210 evaluable participants would yield 60–80 outcome events. With a maximum of 6–8 candidate predictors, this yields an events-per-variable ratio of approximately 10, exceeding the recommended minimum for logistic regression to avoid overfitting. Internal validation will be performed using bootstrap resampling (1,000 repetitions) to estimate optimism-corrected performance. Total enrollment of 300 accounts, with 10% mortality and 20% loss to follow-up, and the understanding that the final evaluable sample will be approximately 210 participants.

### 2.7 Statistical analysis plan

All analyses will be performed using SPSS (Version 27.0) and R (Version 4.4.1).

- Descriptive Statistics: Continuous data will be presented as means ± SD (for normally distributed data) or as medians [IQR] (for non-normally distributed data). Categorical data will be analyzed using Chi-square or Fisher's exact tests.
- Incidence and Survival Analysis: Registry-based incidence and survival rates will be stratified by gestational age, sex, birth weight, and hospital level.
- Predictive Model Development:
  1. Predictor Selection: LASSO regression will be used to identify potential predictors.
  2. Model Building: Multivariable logistic regression will identify independent factors associated with poor neurodevelopmental outcomes.
  3. Model Validation: Internal validation using bootstrap resampling (1,000 repetitions) will assess model performance (discrimination, calibration, and clinical utility).

A two-sided *p*-value < 0.05 will be considered statistically significant.

### 2.8 Safety assessment

- Data Security: All data will be de-identified and stored securely on password-protected servers.
- Adverse Event Monitoring: Adverse events related to clinical care or follow-up will be documented in accordance with institutional protocols.
- Oversight: The Clinical Research Data Monitoring Committee will conduct biannual reviews of study progress and data quality.

## 3 Discussion

This study addresses a critical gap in neonatology and pediatric neurology in China by establishing the Shenzhen Neonatal Stroke Online Registry Database and implementing the nation's first large-scale multicenter, prospective cohort study of neonatal stroke. Consistent with international efforts, such as the Italian Registry of Infantile Thrombosis (RITI), our registry aims to provide continuous, standardized monitoring of aetiology and prognosis (9). While several other countries (6, 7) have successfully established online registries to continuously monitor etiology and prognosis, China currently lacks a dedicated national registry and has not conducted a prospective multicenter study in this field. The establishment of this registry is paramount as it will facilitate the collection of large-volume data, enable sophisticated analysis for quality improvement, support the construction of robust prognostic models, and ultimately promote earlier diagnosis and improved outcomes for neonatal stroke, especially given that neonatal stroke has not yet been incorporated into projections showing the rising incidence and prevalence of adult stroke over the next three decades (16, 17).

The collaboration among the twenty-two participating institutions is a cornerstone of this study, enabling seamless data sharing and ensuring comprehensive, full-cycle neurodevelopmental management from the perinatal period through preschool age. This collective effort will enable continuous monitoring of patient outcomes, ensuring that the diagnosis, treatment, and rehabilitation of neonatal stroke are consistent and well coordinated across institutions.

One of the major contributions of this study is its focus on long-term neurodevelopmental outcomes, addressing a significant knowledge gap in China. International research has shown that between 20% and 50% of pediatric stroke survivors develop long-term cognitive impairments, including language deficits, memory decline, intellectual disabilities, and attention deficits, with some chronic language impairments persisting into adolescence (18, 19). However, these data largely derive from pediatric stroke cohorts that include children beyond the neonatal period. The developmental trajectory and neuroplasticity profile of neonatal stroke differ substantially from later childhood stroke due to the unique vulnerability of the developing brain (e.g., impaired myelination, synaptic pruning abnormalities) and its greater potential for reorganization. Where possible, we will present neonatal-specific outcomes separately. This study represents the first prospective registry-based cohort of neonates with stroke in China, incorporating a biobank of biological samples, including stool and breast milk, and tracking patient outcomes up to 5 years of age. The comprehensive nature of this cohort, which includes detailed neurodevelopmental assessments, will significantly advance standardized management of neonatal stroke nationwide.

We acknowledge that neonatal stroke comprises biologically and prognostically heterogeneous subtypes (arterial ischemic, hemorrhagic, venous sinus thrombosis). Subtype-specific analyses are planned for all primary and secondary outcomes.

The prospective design of this study offers substantial clinical and societal benefits. It is expected to deepen the understanding of neonatal stroke, enhance the management of pregnant women with underlying risk factors, and provide tailored guidance for delivery planning. Furthermore, the protocol mandates early cranial MRI and video EEG testing for infants presenting with postnatal seizures, ensuring prompt and accurate diagnosis. These measures are crucial for the early identification and management of neonatal stroke, facilitating timely interventions and better prognostic outcomes.

Additionally, this study underscores the importance of multidisciplinary collaboration and the critical role of follow-up care in early rehabilitation. By emphasizing early diagnosis, intervention, and long-term follow-up, this research will contribute to a more refined and integrated management system for both perinatal medicine and pediatric care. The anticipated outcomes of this study not only hold promise for reducing the financial and emotional burdens faced by families but also offer significant improvements to national health standards, particularly by enhancing the quality of care for neonatal stroke across China.

However, it is important to acknowledge several limitations of this study. As an observational design, it cannot control for all potential confounding variables, limiting its ability to establish causality. The five-year follow-up period is prone to attrition bias, especially if dropouts are associated with disease severity or socioeconomic factors. Despite standardized protocols, the manual data entry across multiple institutions is vulnerable to inconsistencies and inter-rater variability. Furthermore, the findings may have limited generalizability outside of Shenzhen due to regional differences in demographics, healthcare access, and clinical practices. Moreover, during the 5-year enrollment and follow-up period, MRI protocols, neonatal care standards, and antiepileptic treatment strategies may evolve, potentially influencing outcomes. We will document any major practice changes and adjust analyses accordingly. These limitations should be considered when interpreting the study results and formulating future research directions.

## Supplementary material

Supplementary Material 2

Investigator Declaration.

## References

[1] Dunbar M Mineyko A Hill M Hodge J Floer A Kirton A. Perinatal stroke in premature infants. Pediatr Neonatol. (2021) 62(6):581–2. 10.1016/j.pedneo.2021.06.01734272198

[2] Ferriero DM Fullerton HJ Bernard TJ Billinghurst L Daniels SR DeBaun MR Management of stroke in neonates and children: a scientific statement from the American Heart Association/American stroke association. Stroke. (2019) 50(3):e51–e96. 10.1161/STR.0000000000000183 30686119

[3] Xia Q Guo F Hou X Tang Z Liu L. Perinatal stroke in a Chinese neonatal center: clinical characteristics, long-term outcomes, and prognostic factors. Pediatr Neurol. (2023) 148:111–7. 10.1016/j.pediatrneurol.2023.08.024 37703655

[4] Cole L Dewey D Letourneau N Kaplan BJ Chaput K Gallagher C Clinical characteristics, risk factors, and outcomes associated with neonatal hemorrhagic stroke: a population-based case-control study. JAMA Pediatr. (2017) 171(3):230–8. 10.1001/jamapediatrics.2016.4151 28114647

[5] Dunbar M Kirton A. Perinatal stroke: mechanisms, management, and outcomes of early cerebrovascular brain injury. Lancet Child Adolesc Health. (2018) 2(9):666–76. 10.1016/S2352-4642(18)30173-1 30119760

[6] Kwok T Dineen RA Whitehouse W Lynn RM McSweeney N Sharkey D. Neonatal stroke surveillance study protocol in the United Kingdom and Republic of Ireland. Open Med. (2022) 17(1):1417–24. 10.1515/med-2022-0554 PMC9449691 36128449

[7] Hurd C Livingstone D Brunton K Teves M Zewdie E Smith A Early intensive leg training to enhance walking in children with perinatal stroke: protocol for a randomized controlled trial. Phys Ther. (2017) 97(8):818–25. 10.1093/ptj/pzx045 28789469

[8] Laugesaar R Vaher U Lõo S Kolk A Männamaa M Talvik I Epilepsy after perinatal stroke with different vascular subtypes. Epilepsia Open. (2018) 3(2):193–202. 10.1002/epi4.12104 29881798 PMC5983200

[9] Pelizza MF Martinato M Rosati A Nosadini M Saracco P Giordano P The new Italian registry of infantile thrombosis (RITI): a reflection on its journey, challenges and pitfalls. Front Pediatr. (2023) 11:1094246. 10.3389/fped.2023.1094246 37152311 PMC10159054

[10] Pediatric Neurology Group, Chinese Medical Association. Expert consensus on diagnosis and treatment of pediatric arterial ischemic stroke. Zhonghua Er Ke Za Zhi. (2022) 60(12):1248–52. 10.3760/cma.j.cn112140-20220831-00770 36444424

[11] Boardman JP Ganesan V Rutherford MA Saunders DE Mercuri E Cowan F. Magnetic resonance image correlates of hemiparesis after neonatal and childhood middle cerebral artery stroke. Pediatrics. (2005) 115(2):321–6. 10.1542/peds.2004-0427 15687439

[12] Mercuri E Barnett A Rutherford M Guzzetta A Haataja L Cioni G Neonatal cerebral infarction and neuromotor outcome at school age. Pediatrics. (2004) 113(1 Pt 1):95–100. 10.1542/peds.113.1.95 14702455

[13] Beslow LA Vossough A Dahmoush HM Kessler SK Stainman R Favilla CG Modified pediatric ASPECTS correlates with infarct volume in childhood arterial ischemic stroke. Front Neurol. (2012) 3:122. 10.3389/fneur.2012.00122 23015799 PMC3449492

[14] Lõo S Ilves P Männamaa M Laugesaar R Loorits D Tomberg T Long-term neurodevelopmental outcome after perinatal arterial ischemic stroke and periventricular venous infarction. Eur J Paediatr Neurol. (2018) 22(6):1006–15. 10.1016/j.ejpn.2018.07.005 30249407

[15] Elgendy MM Puthuraya S LoPiccolo C Liu W Aly H Karnati S. Neonatal stroke: clinical characteristics and neurodevelopmental outcomes. Pediatr Neonatol. (2022) 63(1):41–7. 10.1016/j.pedneo.2021.06.017 34509386

[16] Prendes CF Rantner B Hamwi T Stana J Feigin VL Stavroulakis K Burden of stroke in Europe: an analysis of the global burden of disease study findings from 2010 to 2019. Stroke. (2024) 55(2):432–42. 10.1161/STROKEAHA.122.042022 38252754

[17] Wafa HA Wolfe CDA Emmett E Roth GA Johnson CO Wang Y. Burden of stroke in Europe: thirty-year projections of incidence, prevalence, deaths, and disability-adjusted life years. Stroke. (2020) 51(8):2418–27. 10.1161/STROKEAHA.120.029606 32646325 PMC7382540

[18] Xu G Hao F Zhao W Qiu J Zhao P Zhang Q. The influential factors and non-pharmacological interventions of cognitive impairment in children with ischemic stroke. Front Neurol. (2022) 13:1072388. 10.3389/fneur.2022.1072388 36588886 PMC9797836

[19] Heimgärtner M Gschaidmeier A Schnaufer L Staudt M Wilke M Lidzba K. The long-term negative impact of childhood stroke on language. Front Pediatr. 2024;12:1338855. 10.1161/STROKEAHA.120.029606 38774297 PMC11106365

