# Supplementary figures and images for: Online registration of neonatal stroke in Shenzhen: protocol for a multicentre, prospective, observational cohort study
Source: Front Pediatr. 2026 May 8;14:1775052. doi: 10.3389/fped.2026.1775052 (PMC13195399; doi:10.3389/fped.2026.1775052)

Supplementary1 Shenzhen Neonatal Stroke Study Planning Flowchart


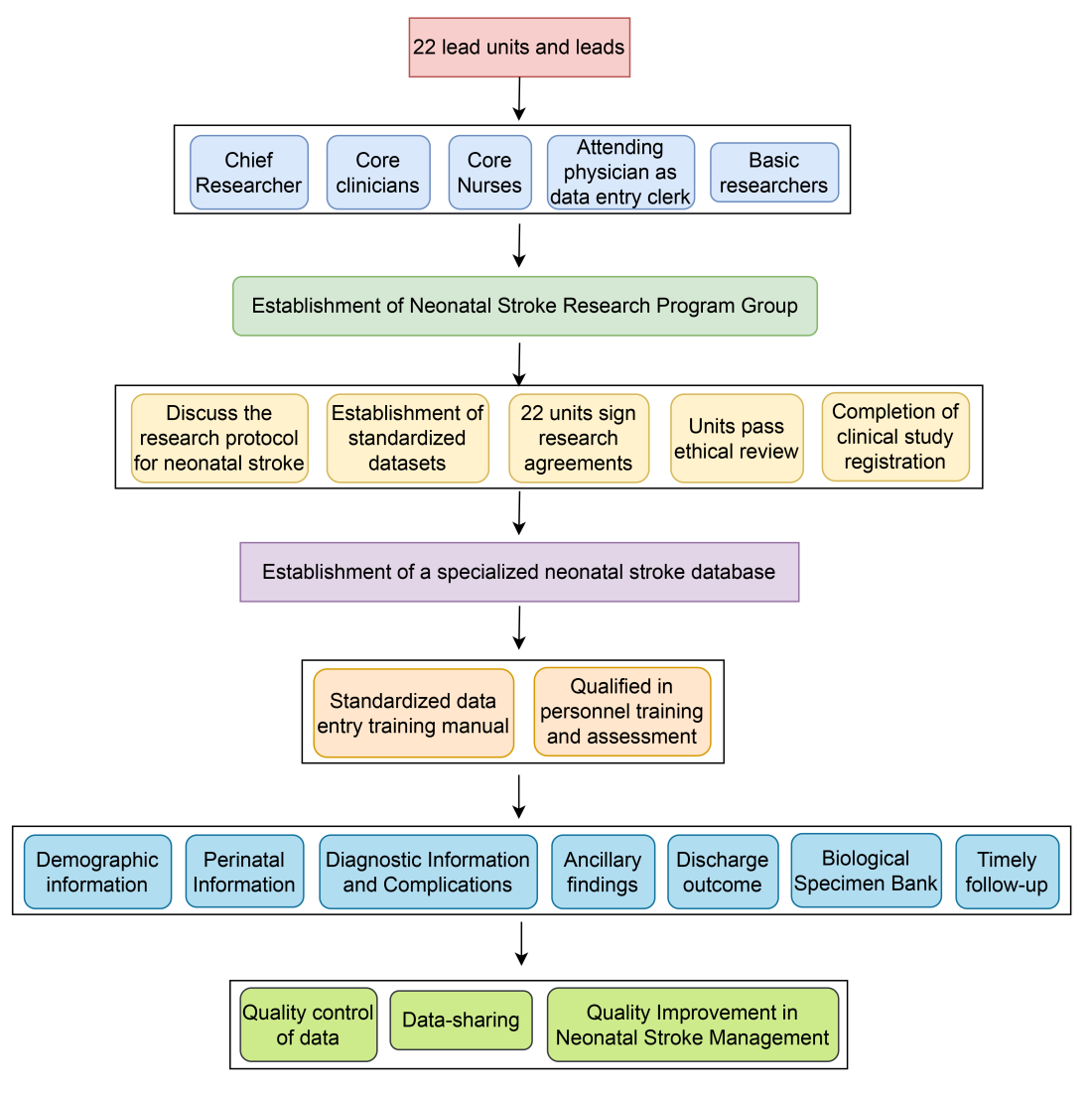

Supplement: Supplementary file 1 [file Supplementaryfile1.docx]
