# Supplementary material for: Online registration of neonatal stroke in Shenzhen: protocol for a multicentre, prospective, observational cohort study
Source: Front Pediatr. 2026 May 8;14:1775052. doi: 10.3389/fped.2026.1775052 (PMC13195399; doi:10.3389/fped.2026.1775052)
Supplement: Supplementary file 2 [file Supplementaryfile2.docx]

| Supplementary Material 2  Information on Variables in the Neonatal Stroke Database |
| --- |
| Instructions for filling out the form  1.Please fill out this form manually to ensure the security of your infant's and guardian's information.  2.Please fill in specific numbers and text descriptions for information such as patient number, hospitalization number, and cranial MRI results.  3.Please click to select gender, mode of delivery and other options.  4.Please specify the time in minutes. |
| 1.Basic Information  Patient number: ____  Patient name: ____  Mother's name: ____  Hospital of birth: ____  Time of birth: ____  Patient Source: □Outpatient Emergency □Delivery admission □Transferred to NICU  Hospital Level: □Tertiary □Tertiary □Secondary  Hospital nature: □Children's Specialty □General Hospital □Maternal and Child Health Center |
| 2. Family history  Hematological disorders: □Yes □No  Congenital heart disease (excluding patent ductus arteriosus): □Yes □No  Autoimmune diseases: □Yes □No |
| 3. Perinatal information  Mother's age: ___  Education level: □Junior high school □High school □College graduate □Post graduate  Mode of conception: □Natural □Test tube baby  Total antenatal care visits: ___  Mode of delivery: □Natural □Forceps or fetal suction vaginal delivery □Cesarean section  Underlying disease: □Cervicitis □Fibroids □Polycystic ovary syndrome  Complications during pregnancy: □Diabetes during pregnancy □Hypertension during pregnancy □Cholestasis  Prolonged bed rest: □Yes □No  Premature rupture of membranes >18 hours: □Yes □No  Placenta: □Placenta praevia □Placental abruption □Placental calcification  Abnormal umbilical cord: □Yes □No  Amniotic fluid: □Normal □I^o^ □II^o^ □III^o^ □Bloody  Apgar score: 1 min ___ 5 min ___ 10 min ___  Umbilical artery blood gas analysis: PH___ BE ___  History of asphyxia resuscitation：□Yes □No |
| 4. Newborn information  Gestational age (w): ___  Birth weight (g): ___  SGA: □Yes □No  Blood glucose (mmol/l):____  Time of discovery of convulsions:____  Seizure presentation:___ |
| 5.Diagnosis and treatment information  Time of discovery of convulsions: ___  Time of admission to NIICU:____  Time of cranial MRI:___ Results:___  Anticoagulation therapy:□No □Yes □Plain heparin □low molecular heparin sodium  Anticonvulsant therapy: □No □Yes □Phenobarbital □Midazolam □Levetiracetam  Antibiotic therapy:□No □Yes □Access □Watch □Reserve  Blood product transfusion therapy:□No □Yes □Fresh frozen plasma □Cryoprecipitation □Thrombospondin Complex □Erythrocyte Suspension  Neurosurgical intervention:□No □Yes □Interventional thrombolysis □Lateral ventricular drainage |
| 6. Auxiliary examination information  Patient full exome result: □Negative □Positive  Father's full exome result: □Negative □Positive  Mother's full exon result: □Negative □Positive (this test is not required, guardian's informed consent is needed)  Coagulation Function Tests: APTT(s):____ PT(s):____ TT(s):____ Antithrombin(%):____ Fib (g/l):____ D-D (ug/ml):____  Complete set of coagulation factors: Coagulation factor II%:____ Coagulation factor V%:____ Coagulation factor VII%:____ Coagulation factor VIII%:____ Coagulation factor IX%:____ Coagulation factor X%:____ Coagulation factor XI%:____ Coagulation factor XII%:____  Autoantibodies:□Negative □Positive  aEEG: Time of examination: ____ Drugs used: □Phenobarbital □Midazolam □Oxcarbazepine□Other  Background: □Normal □Continuous □Discontinuous □Burst suppression □Low voltage  Special events: □Normal □Electrical seizures □Electroclinical seizures  Conclusion: □Normal □Mild □Moderate □Severe |
| 7.Information on complications  Neonatal hypoxic ischemic encephalopathy:□Yes □No  Neonatal sepsis:□No □Yes □Early onset sepsis □Late onset sepsis  Neonatal erythrocytosis:□Yes □No  Neonatal hyperglycemia:□Yes □No  Neonatal hypoglycemia:□Yes □No |
| 8.Biological Sample Registration  Fecal sample number: ___ Time of collection: ___ Storage time: ____  Breast milk sample number: ___ Time of collection: ___ Storage time: ___ |
| 9. Discharge status  Neonatal stroke type: □PAIS □HS □CVST  Length at discharge (cm):____ Weight (kg):____ Head circumference (cm):____  Extrauterine growth retardation: □Yes □No  Treatment outcome: □Cured □Improved □Transferred □In-hospital death  Length of hospitalization (d): ____  Hospitalization cost (yuan): ___ |
| 10.Follow-up examination:  At 6 months of age Survival status：□Died □Alive Lost to follow-up: □Yes □No  Length (cm): ____  Weight (kg): ____  Head circumference (cm): ____  Growth retardation:□Yes □No  Antiepileptic drug therapy:□No □Yes Number of types: ____  Drug use at aEEG:□No □Yes □Phenobarbital □Midazolam □Oxcarbazepine  Background: □Normal □Continuous □Discontinuous □Durst suppression □low voltage  Special events: □Normal □Electrical seizure □Electro-clinical seizure  Conclusion: □Normal □Mild □Moderate □Severe  Bailey 4 Chinese version: Fine motor:___ Gross motor:___ Cognitive:___ Receptive communication:___ Expressive communication:___  At 18 months of age Survival status：□Died □Alive Lost to follow-up: □Yes □No  Length (cm): ____  Weight (kg): ____  Head circumference (cm): ____  Growth retardation:□Yes □No  Antiepileptic drug therapy:□No □Yes Number of types: ____  Brain MRI examination time: ___ Results:___  Drug use at aEEG:□No □Yes □Phenobarbital □Midazolam □Oxcarbazepine  Background: □Normal □Continuous □Discontinuous □Durst suppression □low voltage  Special events: □Normal □Electrical seizure □Electro-clinical seizure  Conclusion: □Normal □Mild □Moderate □Severe  Bailey 4 Chinese version: Fine motor:___ Gross motor:___ Cognitive:___ Receptive communication:___ Expressive communication:___  At 3 years of age Survival status：□Died □Alive Lost to follow-up: □Yes □No  Length (cm): ____  Weight (kg): ____  Head circumference (cm): ____  Growth retardation:□Yes □No  Antiepileptic drug therapy:□No □Yes Number of types: ____  Brain MRI examination time: ___ Results:___  Drug use at aEEG:□No □Yes □Phenobarbital □Midazolam □Oxcarbazepine  Background: □Normal □Continuous □Discontinuous □Durst suppression □low voltage  Special events: □Normal □Electrical seizure □Electro-clinical seizure  Conclusion: □Normal □Mild □Moderate □Severe  Bailey 4 Chinese version: Fine motor:___ Gross motor:___ Cognitive:___ Receptive communication:___ Expressive communication:___  At 5 years of age Survival status：□Died □Alive Lost to follow-up: □Yes □No  Length (cm): ____  Weight (kg): ____  Head circumference (cm): ____  Growth retardation:□Yes □No  Antiepileptic drug therapy:□No □Yes Number of types: ____  Brain MRI examination time: ___ Results:___  Drug use at aEEG:□No □Yes □Phenobarbital □Midazolam □Oxcarbazepine  Background: □Normal □Continuous □Discontinuous □Durst suppression □low voltage  Special events: □Normal □Electrical seizure □Electro-clinical seizure  Conclusion: □Normal □Mild □Moderate □Severe  Bailey 4 Chinese version: Fine motor:___ Gross motor:___ Cognitive:___ Receptive communication:___ Expressive communication:___ |
